# Case Report: Ultrasound-guided microwave ablation combined with postoperative radiotherapy and chemotherapy for advanced tongue cancer

**DOI:** 10.3389/fonc.2025.1564348

**Published:** 2025-07-09

**Authors:** Ting Wei, Jiulong Dai, Tingting Li, Ziyue Hu, Lu Wang, Juan Li, Jie Zou, Jia Feng, Man Lu

**Affiliations:** Department of Ultrasound Medical Center, Sichuan Clinical Research Center for Cancer, Sichuan Cancer Hospital & Institute, Sichuan Cancer Center, University of Electronic Science and Technology of China, Chengdu, China

**Keywords:** ultrasonography, microwave ablation, tongue neoplasms, radiotherapy, drug therapy

## Abstract

**Background:**

While surgery remains the standard treatment for tongue cancer, it is associated with a range of functional and aesthetic sequelae. Additionally, ultrasound-guided microwave ablation (MWA) represents a promising minimally invasive alternative for patients with poor performance status or those unwilling to undergo surgery.

**Case Presentation:**

We report a 50-year-old male patient diagnosed with advanced tongue squamous cell carcinoma and cervical lymph node metastases. Microwave ablation was performed using a power setting of 30 W. Contrast-enhanced ultrasound conducted immediately afterward revealed no enhancement in the ablation area. Following the ablation, the patient received adjuvant radiotherapy and chemotherapy. During the procedure, the patient experienced minimal pain and no significant complications. After more than two years of follow-up, no evidence of metastasis or recurrence was observed, and the patient retained normal speech and swallowing functions.

**Conclusions:**

Ultrasound-guided MWA combined with radiotherapy and chemotherapy offers a safe, effective, and minimally invasive approach to the treatment of advanced tongue cancer.

## Introduction

As a prevalent malignancy in the head and neck region, tongue cancer, which is predominantly squamous cell carcinoma (SCC), continues to contribute substantially to global cancer-related morbidity and mortality (1, 2). Given its essential role in functions such as speech, swallowing, and mastication, preservation of the tongue is critical to maintaining patients’ quality of life (3, 4).

Management of advanced-stage tongue cancer typically consists of surgical resection, most commonly partial or total glossectomy, followed by cervical lymph node dissection and adjuvant radiotherapy (3–5). Although effective in terms of oncological control, these interventions often result in severe functional impairments, including dysphagia, disfigurement, loss of speech, and permanent dependence on feeding tubes, which may lead to considerable psychological distress and social withdrawal (3–5).

In recent years, ultrasound-guided MWA has gained widespread attention as a treatment modality for various malignant tumors, including head and neck cancers (6–9). Ultrasound-guided MWA offers several advantages in the treatment of oral cancers, particularly in anatomically complex regions such as the tongue (6, 8). Real-time ultrasound imaging provides continuous visualization of the tumor and adjacent anatomical structures, enabling precise placement of the microwave ablation needle and real-time monitoring of the ablation zone (6–9). This helps to avoid damage to critical tissues, such as blood vessels, nerves, and healthy mucosa, thereby minimizing collateral thermal injury (6–9). Compared to conventional ablation techniques, MWA also produces higher intratumoral temperatures, larger and more uniform ablation zones, and shorter procedure times, while being less susceptible to the heat-sink effect caused by adjacent blood flow (10, 11). This article presents a case of advanced tongue cancer managed with ultrasound-guided MWA in a patient who declined surgical treatment, and discusses the subsequent application of radiotherapy and chemotherapy to optimize therapeutic outcomes.

## Case presentation

**Figure 1:**
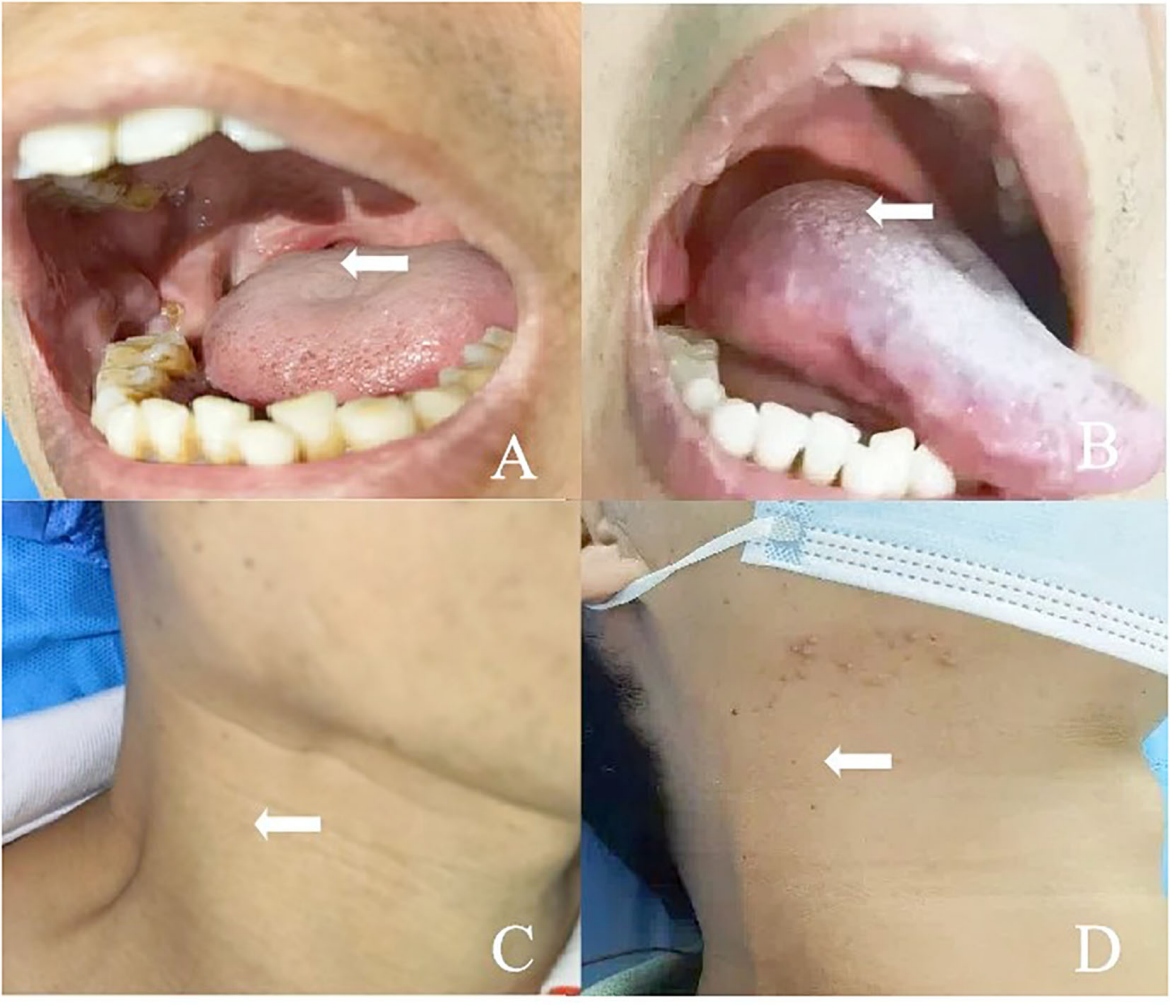
**(A)** A 50-year-old man presented with squamous cell carcinoma of the right tongue base. **(B)** The lesion on the right side of the tongue base disappeared after ablation. **(C)** The patient had right submandibular lymph node metastasis and skin bulge. **(D)** The patient recovered well after ablation of the right submandibular metastatic lymph node.

The patient is a 50-year-old male who discovered a right submandibular mass approximately 3 cm in size. The mass was painless and showed no signs of redness, swelling, or ulceration. It exhibited moderate mobility, firm consistency, and remained stationary during swallowing (**Figure 1**). The patient reported no tinnitus, hearing loss, or trismus. He had previously received oral antibiotic therapy at an outside facility, which led to a slight reduction in the size of the mass; however, throat discomfort persisted. A biopsy of the tongue base mass was performed, and the histopathological analysis confirmed SCC. The patient subsequently presented to our hospital for further evaluation and management. His medical history was notable for chronic gastritis lasting over ten years. He denied any history of malaria, tuberculosis, hypertension, diabetes, or coronary heart disease.

Following multidisciplinary evaluation by the surgical and radiation oncology teams, the patient was advised to undergo an extended glossectomy and cervical lymph-node dissection for definitive management of tongue SCC. The procedure was associated with a risk of postoperative functional deficits, such as impaired speech, chewing, and swallowing. In light of these potential quality-of-life consequences, the patient expressed reservations about conventional surgery. The interventional team therefore presented ultrasound-guided MWA as an alternative modality, outlining its feasibility, potential benefits, and attendant risks in detail. After demonstrating full understanding, the patient provided written informed consent and elected to proceed with MWA in lieu of surgical resection.

## Pre-ablation assessment

Ultrasound examination (gray-scale ultrasound and contrast enhanced ultrasound (CEUS)) was performed by a radiologist (M.L.). The ultrasound probe used was a 5–12 MHz transducer (Philips EPIQ 7 ultrasound system, Bothell, WA, USA).

The patient was positioned in a supine position and local anesthesia (10 mL of lidocaine hydrochloride gel) was administered to alleviate pain. Gray-scale ultrasound and color Doppler ultrasound (CDFI) were used to assess the size, shape, boundary, location, blood flow, and adjacent tissues of the tongue tumor and cervical lymph nodes. CEUS was performed by intravenously injecting 2.4 ml of Sonovue (Bracco, Milan, Italy), followed by a 5 ml saline flush. The CEUS was used to evaluate the active components within the lesions and plan the ablation path.

**Figure 2:**
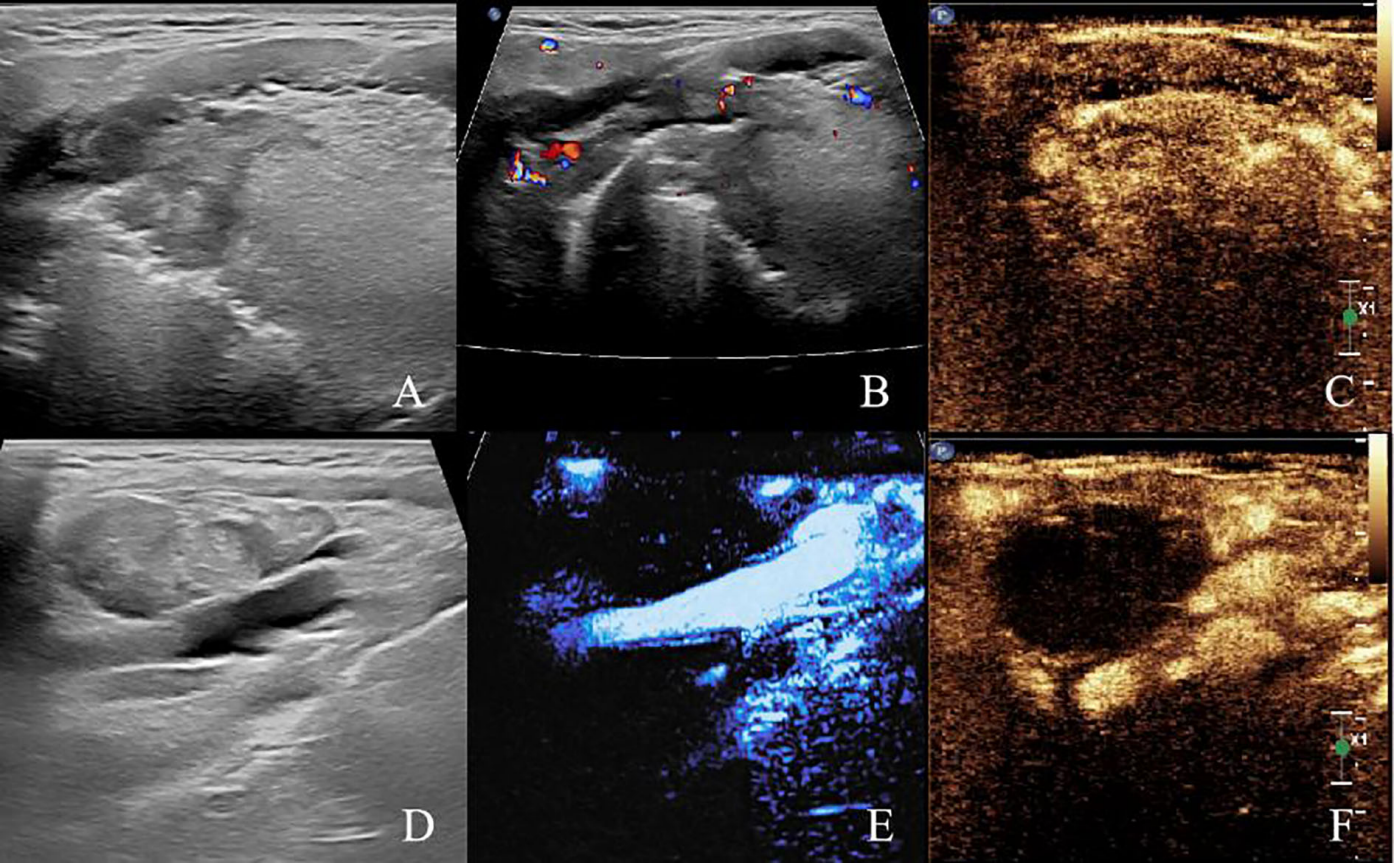
Pre-ablation submandibular ultrasonography **(A)** Two-dimensional ultrasound image of the right tongue base tumor. **(B)** CDFI image of the right tongue base tumor. **(C)** Preoperative CEUS image of the right tongue base tumor. **(D)** Two-dimensional ultrasound image of right cervical lymph node (level II). **(E)** CDFI image of right cervical lymph node (level II). **(F)** Preoperative CEUS image of right cervical lymph node (level II).

Two-dimensional ultrasound revealed a hypoechoic lesion approximately 28x19 mm in size located in the right side of the base of the tongue. The lesion had unclear borders, irregular shape, and heterogeneous internal echoes. CDFI showed the presence of punctate and linear blood flow signals within the lesion. In the right cervical region (level II), an enlarged lymph node measuring approximately 35x19 mm was identified. The cortex appeared thickened with increased echogenicity, the cortico-medullary junction was indistinct, and the parenchyma showed heterogeneous echotexture. A large hypoechoic fluid was observed, and CDFI revealed punctate and linear vascular signals within the solid component. The enlarged lymph nodes abutted the internal jugular vein, with evidence of compression, adhesion, and indistinct margins. CEUS showed that the lymph node in the right cervical region exhibited rapid and high enhancement during the arterial phase, with areas of no enhancement, and rapid washout during the venous phase. The hypoechoic mass at the base of the tongue showed rapid, high enhancement during the arterial phase and rapid washout in the venous phase (**Figure 2**).

MRI showed an irregular soft tissue mass in the right side of the base of the tongue, with a high suspicion for malignant tumor. Enlarged necrotic lymph node was observed adjacent to the right carotid sheath.

Pre-ablation tests included complete blood count, liver and renal function tests, and coagulation tests (prothrombin time and activated partial thromboplastin time). Additional examinations included pulmonary function tests, electrocardiogram, echocardiography, and fiberoptic laryngoscopy.

## Ablation methods

This procedure was approved by the Ethics Committee of Sichuan Cancer Hospital (grant number SCCHEC-03-2017-009).

### Lymph node ablation

MWA was performed by the same experienced radiologist (M.L.). The MWA instrument (KY-2000; Kangyou Medical, Nanjing, China) was used to administer microwave energy.

**Figure 3:**
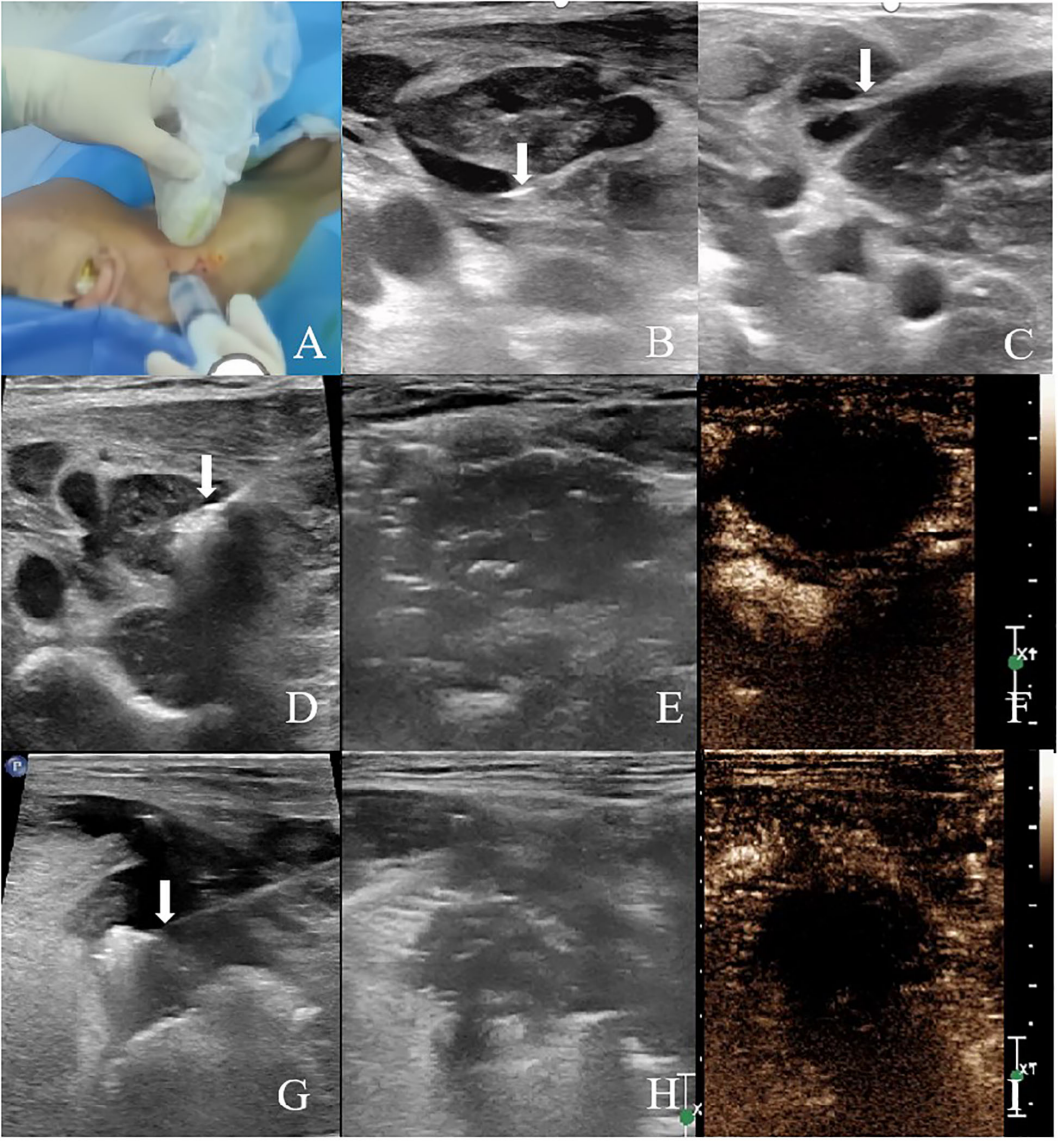
Ultrasound-guided MWA and immediate postoperative CEUS **(A–C)** Hydrodissection with normal saline was performed prior to MWA to isolate the target lesion. Arrows indicate the tip of the needle. **(D)** Ultrasound image of MWA of the right cervical lymph node (level II). **(E)** Two-dimensional ultrasound image of the right cervical lymph node (level II) after ablation. **(F)** CEUS was performed immediately after ablation, and there was no enhancement in the arterial phase and venous phase of the ablated area of the right cervical lymph node (level II). **(G)** Ultrasound image of MWA of the right tongue base tumor. **(H)** Two-dimensional ultrasound image of the right tongue base tumor after ablation. **(I)** CEUS was performed immediately after ablation, and there was no enhancement in the arterial phase and venous phase of the ablation area of the right tongue base.

The patient was in a supine position with the head tilted toward the healthy side. After cervical plexus nerve block and local anesthesia, the hydrodissection technique was used to separate the lymph node from the adhered internal jugular vein and surrounding tissues (**Figure 3**). After aspirating the fluid from the lymph node, the ablation needle was precisely placed at the base of the lymph node under ultrasound guidance. Ablation was performed from the bottom upwards, layer by layer, at a power of 30W for 13 minutes (**Figure 3**). Post-ablation, CEUS showed no enhancement in the arterial and venous phases of the ablated lymph node (**Figure 3**).

### Tongue tumor ablation

Prior to the ablation of the tongue lesion, glossopharyngeal nerve block and local anesthesia were performed to alleviate pain during the tongue base ablation procedure. A needle was inserted from the right submandibular area. Normal saline was injected subcutaneously to protect the skin, and ice-cold saline-soaked gauze was used to protect the tongue and surrounding oropharyngeal tissues. Under ultrasound guidance, the ablation needle was accurately placed at the base of the mass. A movable ablation technique was employed, with ablation performed from bottom to top and from inside to outside, using a power of 30W for 12 minutes (**Figure 3**). Post-ablation, CEUS showed no enhancement in the arterial and venous phases of the ablated tongue lesion (**Figure 3**).

## Intraoperative pain assessment

Pain assessment was conducted using the VAS (Visual Analog Scale). Intraoperative pain was rated at 1, postoperative pain at 8 hours was rated at 3, and after applying ice, the pain was relieved. The pain score was 0 the following morning.

## Radiation therapy and chemotherapy

After the ablation, the patient underwent radiation therapy and chemotherapy. The total radiation dose was 72 Gy, along with six cycles of chemotherapy (one cycle every 21 days).

## Postoperative follow-up

**Figure 4:**
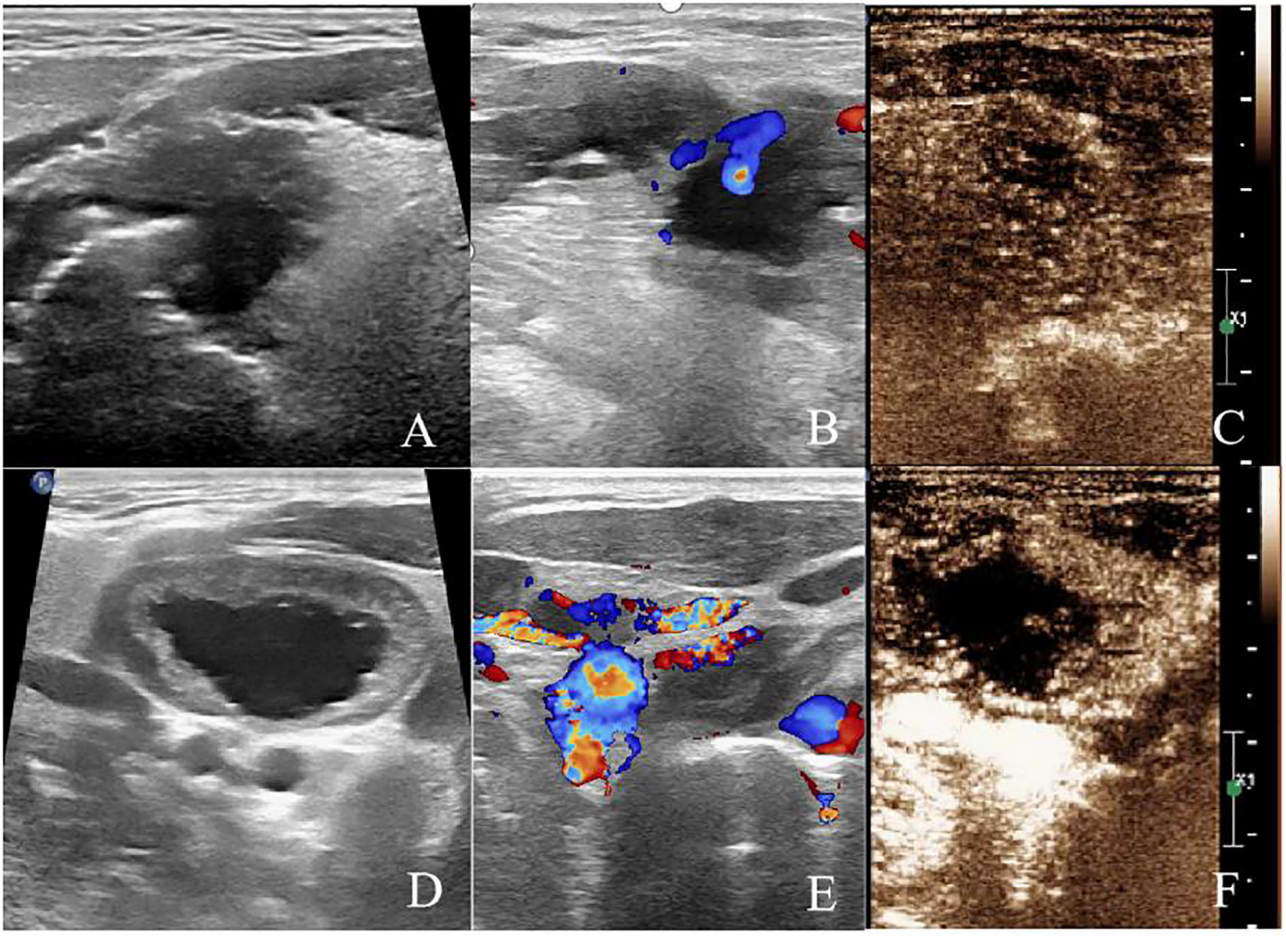
After 2 months of ablation. **(A)** Two-dimensional ultrasound showed hypoechoic area in the ablation area of the tongue base. **(B)** CDFI showed no blood flow signal in the ablation area of the tongue base. **(C)** CEUS showed no enhancement in the ablation area of the tongue base. **(D)** Two-dimensional ultrasound showed hypoechoic area in the right cervical lymph node (level II). **(E)** Microvascular imaging showed no blood flow signal in the right cervical lymph node (level II). **(F)** CEUS showed no enhancement in the ablation area of the right cervical lymph node (level II).

The patient was followed up regularly with ultrasound examinations and clinical assessments. Two months later, follow-up imaging showed no significant blood flow signals in the tongue lesion or cervical lymph node on microvascular flow imaging. CEUS revealed no enhancement in either the arterial phase or venous phase within the ablated areas. At five months, re-evaluation showed gradual resorption of the ablated lesions, with no enhancement observed on CEUS in either the tongue lesion or cervical lymph node. At nine months, the tongue lesion had completely resolved, and the ablated cervical lymph node had decreased in size. CDFI and microvascular flow imaging showed no detectable blood flow, and CEUS demonstrated no enhancement in either the ablated tongue area or cervical lymph node, indicating complete ablation. The patient has been followed up regularly for two years, with no evidence of recurrence or metastasis (**Figure 4**).

## Discussion

This case report presents an advanced tongue SCC patient who declined conventional surgical resection due to the potential postoperative functional impairments, including compromised speech, mastication, and swallowing. Instead, the patient underwent ultrasound-guided MWA combined with chemoradiotherapy, achieving satisfactory local tumor control while preserving critical oral functions. To our knowledge, this represents the first application of this multimodal approach for advanced tongue cancer, highlighting a novel and less invasive therapeutic option for patients contraindicated for or unwilling to undergo extensive surgery.

Standard management of advanced tongue SCC typically involves radical surgical excision with cervical lymph node dissection followed by adjuvant radiotherapy and chemotherapy. While effective oncologically, these interventions often result in substantial postoperative morbidity, including functional deficits and facial deformities that significantly reduce quality of life. Total glossectomy, in particular, can cause irreversible loss of speech and swallowing abilities, which profoundly affect psychological well-being and social integration (3, 4).

MWA offers a promising solution to these challenges. As demonstrated in this case report, MWA can effectively ablate both the primary tumor and metastatic lymph nodes. Real-time ultrasound guidance during the MWA process ensures precise tumor localization and decreases the likelihood of incomplete ablation while minimizing damage to surrounding healthy tissues. It is vital for tumors located near critical structures like nerves and blood vessels, as damage to these areas could lead to significant functional impairments. The minimally invasive nature of MWA preserves the essential functions of the tongue, such as speech, swallowing, and chewing. It reduces the risk of complications commonly associated with major surgeries, such as bleeding, infection, and wound healing issues. Compared to surgical procedures, the recovery time after MWA is much shorter, and patients typically return to normal activities within a few days. Furthermore, pain associated with the ablation procedure is significantly reduced, resulting in lower levels of discomfort during and after the procedure compared to traditional surgery.

The patient, in this case, refused surgery due to concerns about functional impairments, particularly speech and swallowing difficulties, which are often irreversible after total glossectomy. By opting for ultrasound-guided MWA, the patient could effectively treat the tumor while preserving the functional integrity of the tongue. Moreover, the combination of MWA with postoperative radiotherapy and chemotherapy helped control residual tumor cells and prevent a recurrence. Follow-up imaging and clinical examinations showed no evidence of tumor recurrence. In terms of quality of life, the patient had a good recovery. Postoperatively, the patient’s tongue function was well-preserved, and both speech and swallowing abilities were normal, which is crucial for the patient’s social and psychological well-being. Unlike the potential permanent disfigurement and functional loss associated with surgery, the patient was able to maintain a high level of quality of life.

Despite the promising outcome reported here, several limitations must be acknowledged. This is a single-case report with a relatively short follow-up period, which limits the generalizability of the findings. Future prospective studies with larger patient cohorts and extended follow-up are essential to validate the safety, efficacy, and durability of this combined treatment modality and to better define its role relative to conventional therapies.

## Conclusion

In summary, this case underscores the potential of ultrasound-guided MWA combined with chemoradiotherapy as a feasible and function-preserving alternative to radical surgery in patients with advanced tongue cancer.

## Data Availability

The original contributions presented in the study are included in the article/supplementary material. Further inquiries can be directed to the corresponding author.
